# The interplay between structure and agency in shaping the mental health consequences of job loss

**DOI:** 10.1186/1471-2458-13-110

**Published:** 2013-02-06

**Authors:** Julia Anaf, Frances Baum, Lareen Newman, Anna Ziersch, Gwyneth Jolley

**Affiliations:** 1 Southgate Institute for Health, Society and Equity, Flinders University, Room 248 Health Sciences Building, GPO Box 2100, Adelaide, SA, 5001, Australia; 2 Southgate Institute for Health, Society & Equity & SACHRU, Flinders University, Health Sciences Building Room 2.05, GPO Box 2100, Adelaide, SA, 5001, Australia; 3 Southgate Institute for Health Society & Equity, Flinders University, Room 2.55, Health Sciences Building, GPO Box 2100, Adelaide, SA, 5001, Australia; 4 Southgate Institute for Health, Society & Equity, Flinders University, Room 253, Health Sciences Building, GPO Box 2100, Adelaide, SA, 5001, Australia; 5 South Australian Community Health Research Unit, Flinders University, GPO Box 2100, Adelaide, SA, 5001, Australia

**Keywords:** Job loss, Unemployment, Mental health, Agency, Structure, Mitsubishi, Redundancy, Automotive, Manufacturing

## Abstract

**Background:**

Job loss is a discrete life event, with multiple adverse consequences for physical and mental health and implications for agency. Our research explores the consequences of job loss for retrenched workers’ mental health by examining the interplay between their agency and the structures shaping their job loss experiences.

**Methods:**

We conducted two waves of in-depth, semi-structured interviews with a sample of 33 of the more than 1000 workers who lost their jobs at Mitsubishi Motors in South Australia during 2004 and 2005 as a result of industry restructuring. Interviews capturing the mental health consequences of job loss were recorded and transcribed verbatim. Thematic analysis was employed to determine the health consequences of the job loss and the impact of structural factors.

**Results:**

Main themes that emerged from the qualitative exploration of the psychological distress of job loss included stress, changes to perceived control, loss of self-esteem, shame and loss of status, experiencing a grieving process, and financial strain. Drawing on two models of agency we identified the different ways workers employed their agency, and how their agency was enabled, but mainly constrained, when dealing with job loss consequences.

**Conclusions:**

Respondents’ accounts support the literature on the moderating effects of economic resources such as redundancy packages. The results suggest the need for policies to put more focus on social, emotional and financial investment to mediate the structural constraints of job loss. Our study also suggests that human agency must be understood within an individual’s whole of life circumstances, including structural and material constraints, and the personal or interior factors that shape these circumstances.

## Background

This article explores the mental health consequences of job loss resulting from the downsizing and partial closure in 2004 and 2005 of the South Australian subsidiary of Mitsubishi Motors, a major automotive manufacturing company. Job loss is a discrete life event with multiple adverse consequences for physical and mental health, including depression and impaired psychosocial functioning [1]. Mental health results from the complex interplay between biological, psychological, social, environmental and economic factors [2]; while social environments incorporate material, behavioural and psychosocial factors that all strongly influence health [3]. Reviews of literature [1,4] show that individuals experiencing job loss may be the victims of external economic and social forces, and their scope to exercise agency in support of their health is strongly constrained by these structural forces: an interplay that is explored in our research.

### Job loss and mental health

Job loss is the termination of a worker’s employment in response to planned closure or company downsizing, and is linked to economic change, company organisation, or increased use of technology [5]. Under competitive capitalism job losses are now more often unexpected events due to company failure rather than planned restructuring [6]. A wide literature documents the impact of job insecurity, job loss and unemployment on physical and mental health [7]. There is a nexus between these three discrete areas of research as many workers experience job insecurity prior to job loss which is a life event that ends in unemployment unless a new job is obtained immediately [8].

Job insecurity is most often conceptually linked with the probability of involuntary job loss due to retrenchment or when jobs become redundant [9]. It is a chronic health stressor experienced from the stage of threatened unemployment and heightened in industrialised countries since the 1990s due to economic and labour market changes [10,11]. Forty per cent of Australian jobs are now non-permanent, with debates on the extent to which these are precarious [12]. Anticipation of insecurity and major organisational change results in increased self-reported morbidity [13] which may begin as soon as workers learn that jobs are in jeopardy [14]. The threat of redundancy is potentially equal to, if not greater than, the actual event. An Australian study on job insecurity [15] showed that certainty of job status, even the certainty of redundancy, may be less psychologically detrimental than prolonged insecurity.

Research on employees’ emotional stages in dealing with downsizing and closure has been linked to stages of grieving which include denial, anger, bargaining, depression, exploration and acceptance [16,17]. Job loss usually involves a sequence of stressful events or stages, including anticipation through to actual layoff, job search, training, and finally re-employment [18] within the context of economic change [19]. Manufacturing industry workers face longer periods of non-employment than those in primary industries, with workers tending to be, on average, lower skilled, with longer job tenure and from larger firms than other industries; factors compounding difficulties for re-employment [20] p. 329.

Job loss affects the interaction between individual behaviours, cognitions and emotions, and the material and social contexts of life, with the resulting psychosocial environment influencing positive self-regard and productivity [21,22]. The health impacts of job loss are more difficult to assess within the contemporary labour market where workers in so-called ‘permanent’ jobs still experience insecurity from being increasingly subject to, or aware of, the potential for restructuring and downsizing [23]. Many workers who lose their jobs now find limited, intermittent and more health damaging forms of work that are ‘contingent’ [24]. These involve short tenure, low wages, poor statutory entitlements, job insecurity and curtailed social benefits [25]. They are features of an employment continuum that includes unemployment, economically inadequate employment, and economically adequate employment that may still not be optimal in either psychological or economic terms [26].

Psychosocial and other working conditions are generally worse under precarious employment, with different patterns of adverse occupational exposures identified between groups of precariously employed workers [23]. The most frequent and prominent outcomes of becoming unemployed are symptoms of psychiatric disorder and distress, particularly depression [27]. This can rob life of pleasure and lead to cognitive, motivational and behavioural consequences and neuro-chemical changes [28]. Job loss is high on the list of stressful life events [29], triggered by external stressors including economic downturns and ‘layoffs’, as well as internal stressors including fear of failure [28]. Stressful events may influence physical and mental health and may be additive, with the adverse consequences of losing a job resulting in a potential ‘chain of adversity’ [1] p. 39. This has implications for older workers who are often at greater risk of job loss [9,12,20] and face problems of discrimination, lack of self-esteem and the level of retraining necessary due to redundant skills [30].

Job loss harms health because it represents a serious negative life event with important economic consequences [31], including a lower probability of subsequent employment and considerably reduced future wages and earnings. Associated financial stress is often chronic and experienced as uncontrollable, and is defined as ‘the unpleasant feeling that one is unable to meet financial demands, afford the necessities of life and have sufficient funds to make ends meet’ [28] p. 4. Financial and psychological strain following job loss may undermine the resources needed to cope with other adverse life events [32,33]. Strategies for coping with the range of stressors may also be harmful, with suicidal behaviour perhaps the most extreme [34-36].

Perceived control or ‘the belief that one can determine one’s own internal states and behaviour, influence ones’ environment, and / or bring about desired outcomes’ critical to mental health is influenced by job loss and job insecurity [37]. Perceived control protects against stress and has positive biological effects [38], with a series of cross sectional studies showing the relationship between low perceived control and poor health [39,40]. Financial stress leads to decreased levels in reported feelings of control that may in turn lead to increased distress [41].

Employment provides not only secure income and socioeconomic status but socialisation, personal growth, opportunities for engaging in social networks and the ‘experience of self in a core social role’ [21] p. 99. When people are denied status and respect they are vulnerable to feeling inferior and worthless [12]. Grief or a grieving process may therefore be another outcome of job loss, as it is part of a range of feelings accompanying loss of status and respect. Grief is defined as ‘a neuropsychobiological response to any kind of significant loss with elements typical and unique to each individual or situation’ [42] p. 533. Job loss due to industry restructuring arguably involves both elements as it is experienced as a ‘private trouble’ for each individual, as well as a ‘public issue’ as a widely shared experience [43]. It is important for retrenched workers to mourn the loss of a job, as they may require help to cope with potential multiple losses [44]. These include the roles of worker and family provider, the ‘work family’, a sense of being productive, social status, pride and dignity, and self-esteem. Associated emotions including shame, anger, guilt and shock may be experienced in response [45].

A review of job loss and unemployment [4] includes research supporting a view that reactions to job loss are contingent on the moderating effect of economic resources in the causal chain between job loss and its effects [46], and that ‘availability of income may be the most important determinant of the expression of psychological and health symptoms’ [47] p. 50. Financial strain and its consequences are the ‘critical mediators in the relationship between unemployment and depression’ [1] p. 303. Financial strain mediates the relationship between unemployment status and depression while subsequent re-employment helps lower the influence of financial strain on depression [48].

The health consequences of job loss are grounded in structures that have implications for retrenched workers’ agency, as discussed in the following section.

### An agency and structure approach: two typologies of agency

The term ‘agency’ generally refers to purposive human action or behaviour, decision-making strategies and exercise of choice in pursuing personal aims. It raises issues of motivation and the personal and cultural resources that facilitate a capacity to act [49]. Agency is mediated by socio-economic structures and the skills, motivations, and both interior and material resources available to each individual. These are influenced by educational opportunities and attainment, family supports, financial resources, employment status, and personal subjectivities including gender, age, race, dis / ability and sexuality.

Theoretical insights from two models of agency devised by Lister [50] and Hoggett [51] provide nuanced agency perspectives for understanding responses to job loss. Lister’s typology situates constraints to agency and participation within the context of existing social relations; highlighting the different actions individuals employ to overcome poverty or disadvantage. At the individual level these include (just) ‘getting by’, and more strategic ‘getting out’ (of) responses. At the collective or political level these are everyday ‘getting back’ (at) responses by individuals against ‘the system’, while ‘getting organised’ involves more strategic engagement at the community or political level to improve the well-being of the wider community.

Hoggett’s agency typology identifies that constraints to agency are also rooted in people’s unique interior selves and their fluctuating levels of reflexivity. It acknowledges the reality that individuals sometimes lack insight into things they may say or do in response to a range of emotional or life events [such as job loss], and the potential impact this has on the self or others. Hoggett’s agency typology emphasises ‘reflexive’ agency, mediated by a range of triggers that are grounded in material disadvantage, past experiences within diverse social relationships, and from dealing with a wide range of enabling and constraining structures [51] p. 48.

In Hoggett’s model individuals are recognised as ambivalent and emotionally driven, with an unconscious subjective dimension: social subjects with agency but unable to always exercise it reflexively [52]. Hoggett’s model spans high levels of reflexivity through to habitual or instinctive behaviour or non-reflexivity. It also acknowledges a range of agency perspectives on a continuum from the creative ‘self-as-agent’ to the passive ‘self-as-object’. Hoggett [51,53] challenges agency definitions confined to rational behaviour that exclude non-rational actions often involving unintended consequences. Without recognising the importance of complex subjectivity and these forms of agency there is no adequate explanation for addictions, depression, or self-harming behaviours, unless attributable to genetic or physiological factors. Both the interior world of emotional suffering and the external world of structural constraints impact on the individual and his or her capacity for agency, and in turn, the ability to take actions to either promote or detract from health.

While job loss is understood to be detrimental to mental health, less is known about how structural factors affect the way workers respond to losing their jobs. We contribute to this understanding by examining the mental health impacts of job loss and the ways in which workers’ experiences of job loss from one automobile factory were shaped by the company and public policies prevalent at the time of the job loss. Lister’s and Hoggett’s agency models are used to theorise the interplay of agency and structural factors.

## Methods

We draw on two waves of in-depth, semi-structured interviews with a sample of 33 of the more than 1000 workers who lost their jobs at Mitsubishi Motors in South Australia during 2004 and 2005. This qualitative research was nested within a two-year longitudinal study of 371 of the workers, examining the impacts of retrenchment from automotive plant downsizing and closure in southern Adelaide which resulted in 700 forced, and 400 voluntary, retrenchments across two Mitsubishi automotive plants. The larger surveyed sample was recruited through a mass mail out to workers, visits to the factory sites, and snowball sampling. The research was designed to collect three waves of survey data and two waves of semi-structured in-depth interviews: with only qualitative data reported here. Further aspects of this study included the impact of job loss on children [54]; on the wider community [55]; and contextualised by the prevailing policy environment [56]. Ethics approval was granted by the Flinders University Social and Behavioural Research Ethics Committee prior to commencement.

As part of the first survey, respondents were asked if they would participate in an in-depth interview about their job loss and 264 agreed. From these, 40 were interviewed in a first round of interviews and 33 completed two in-depth interviews each about their job loss experiences. This paper is based on the experiences of these thirty-three. The interviews were recorded and transcribed verbatim. Framework analysis [57] was employed for the interview data analysis. This involves five steps spanning familiarisation, or gaining a broad overview through ‘immersion’ in the data; reading transcripts and documents and listing key ideas and themes; identifying analytical categories by devising a thematic framework based on the questions from the interview topic guide; coding of *a priori* and emerging themes using Nvivo software; charting, or taking data from its original context and rearranging it into charts laid out in the best form for writing up the study; and mapping and interpretation; or reviewing themes, comparing experiences, looking for patterns and connections [57].

## Results

Here we present results including the respondents’ demographic profile, the state of their mental health, and the ways in which they felt job loss affected them both psychologically and materially.

### Demographic profile

The interview sample comprised 27 men and six women, reflecting the gender representation in the Mitsubishi workforce. Their median age was 47.4 years with an age range from 29 to 63 years. The median employment term at Mitsubishi was 19 years, spanning one to 39 years. Workers held diverse positions ranging from managerial to process or production work. One respondent earned more than $130,000; three earned between $78,000 and $130,000; nine between $52,000 and $78,000; ten between $41,000 and $52,000; and ten below $41,000. This compared with average wages in Metropolitan Adelaide in 2004 which were $37,000 [58].

### Mental health

We asked workers about the psychological implications of job loss and a small number spoke of positive changes. Two workers no longer felt the stress of uncertainty, another two were able to leave the poor working conditions in the foundry, two found new jobs immediately, and three spoke of flexibility and more time to spend with children. These results support a review by Hanisch [4] showing that job loss may eventually be viewed positively by some workers as it can facilitate a change of career and life direction [59,60], as well as leaving unchallenging or unsatisfying employment, building upon competencies, and re-evaluating career goals and priorities [60]. However, many respondents reported a range of negative feelings and psychological outcomes including depression and, at the extreme end, suicidal ideation experienced by two workers.

As Tracy explained, workers had been living with uncertainty for an extended period of time prior to job loss. She claimed:

"It’s just a very depressing place there sometimes … From the day I started people were telling me it was finishing up now, that was eight years before it shut down. (Tracy, aged 31, production worker)"

Some workers who told of extreme distress indicated that they were possibly experiencing clinical depression. Doreen, whose husband had also worked at Mitsubishi, stated:

"My husband is not very well and he doesn’t do much at all. He’s depressed, so he’s very hard to motivate… With depression people sink into black holes and they can’t do anything…He took [redundancy] really badly because he had been there 37 years and he was home ill [at the time of the announcements]… No-one contacted him until I’d say about five or six months afterwards … [and] that made the depression worse … They [Mitsubishi] were supposed to send a counsellor to him and they never did. (Doreen, aged 59, canteen worker)"

Raymond spoke of suicidal behaviour when responding to a question about whether redundancy had affected his mental health. He claimed:

"Yeah, mentally it has - it was there from the start, but I sort of kept ignoring until it gradually got to the stage where it just blew up, where I had to tell somebody. That’s when I told my local doctor and that and that’s when [s/he] sent me to a shrink and when the police came around to pick up the rifles… I was suicidal because I was starting to get depressed with not earning as much as I used to and not being able to pay the bills and that. (Raymond, aged 51, maintenance worker)"

Angas, a second worker who experienced suicidal ideation, identified a further dimension to the issue of counselling availability raised by Doreen. He argued that his emotional state was so precarious that it precluded him from making even positive choices in support of his health. These included accessing available post-retrenchment material supports, and access to counselling made available to workers. As Angas explained:

"I’ve been so stressed that the support services might be there, but because of my circumstances I’m just off the planet in the sense like I’m trying to deal with myself, sort of getting myself together kind of thing and I don’t think people realise what this does. It’s soul destroying and you’ve got to pick yourself up …[then] You’ve got to pay your rent and stuff like that and people don’t realise that it’s not a small thing. (Angas, aged 55, tradesman)"

Angas’ is one account highlighting why it may sometimes be extremely difficult for an individual to exercise creative agency in support of his or her health, as agency is bound up with internal conflicts as well as structural constraints [51]. Angas’ explanation suggests that he is just ‘getting by’ under Lister’s agency typology; engaging his agency to meet just basic needs. He also alludes to experiencing *social suffering*[61] encompassing the interior world of psychic suffering and the external world of structural oppression [52]. For although Angas had been the recipient of a relatively generous redundancy package tied to his years of service, this alone did not mediate the psychological consequences of job loss. He explained: 

"Since then I became - at some stage there, because of the circumstances, I did become suicidal and how can I put it? When I got the money [redundancy package] I mean I was sitting down twiddling my thumbs wondering what I was going to do with myself."

Respondents who gave accounts of distress or low levels of mental or psychological health associated this with experiencing stress, changes to their perceived control, loss of self-esteem, shame and loss of status, grief or a grieving process, and financial strain; factors that are explored in the following sections.

### Stress

Stress is a ‘state of mental, emotional, or other strain’ [62] p. 85. Most respondents claimed to have experienced stress from job loss. They linked this to ‘all the guys leaving’, experiencing ‘roller coaster emotions’, ‘leaving a comfort zone’, and from having a ‘draining and emotional’ worker support role. Reflecting the literature on the stress associated with job insecurity and the threat of redundancy [10,14,63] Raymond noted: 

"[The media] were putting rumours in saying they were closing down before [the CEO] had a chance to publicly tell everyone and I think that hurt a lot because a lot of people were scared, and then all of a sudden when he did announce it, it hit a lot of people very emotionally … it hit hard, and some very hard. (Raymond, aged 51, maintenance worker)"

Douglas, a general manager aged 58, told of the stress of uncertainty generated by on-going media speculation about rumoured downsizing and closure and claimed:

"I suppose the uncertainty over the years of rumours, discussion about whether or not we were going to succeed, because every time that came up our sales would drop and so a track of that over the years shows any uncertainty cost us sales. …sometimes it’s difficult too with overseas management making decisions in a global sense that affect us."

Doreen claimed to have experienced stress from bearing the brunt other workers’ heightened emotions, and from:

"saying goodbye to all the men. It was dreadful. There were some that were very happy to go. And then there were people, men my age … You knew they’d never work again. Some of them were crying. Some of them were really angry. You know, saying that they had lost the choice of working till they were 65. It had been taken away from them. How dare people do that to them? Then there were people going straight from there into another job, so they were ecstatic. It was just a whole range of different emotions. And that was, in my opinion, it was pretty horrible. (Doreen aged 59, canteen worker)"

Doreen’s account highlights her own stress, but also the other workers’ negative emotions including shame, anger, guilt and shock [45]. It also notes that positive feelings are sometimes experienced by those who see job loss as an opportunity rather than as a threat. This diversity of seemingly contradictory emotional responses to the same life event of job loss was displayed quite openly within the more informal canteen setting.

Certain roles at Mitsubishi emerged as being particularly stressful, with negative emotional outcomes. Rosslyn, facing her own redundancy while supporting others through the implementation process as part of her employment role, stated:

"I must admit that I’ve been up and down and some days I’ve been quite teary … Because you know, on some days, it sounds awful, but some days, if anybody sort of yelled at me or I did something wrong, I’d have to go. Because I’d be in tears and you hate it, you really do. You feel that you’re out of control. (Rosslyn, aged 45, administrative assistant)"

In her worker support role Rosslyn was both the target of, and responsible for dealing with other workers’ grief and anger, and these projected emotions had touched an aspect of her interior self that at times had made it all too much to bear. The stress of supporting others while also being concerned and anxious about her own future job prospects took a toll in ways Rosslyn felt she did not fully understand. She claimed:

"You find sometimes that you are sort of lashing out in a way, and you don’t mean to, but you do. And being aware of that… Just different emotions and that. And you sort of wonder now, is this the job? Am I anxious? What am I worried about? …and sort of being conscious and aware of that."

Raymond, who was also responsible for supporting retrenched employees throughout the redundancy process, claimed to have been affected ‘emotionally and psychologically’. He stated that he too had ‘lashed out’, and that he had to ‘hold it [his emotions] back’ because:

"I couldn’t be seen to be upset because I had to try and be there for other workers … Well I tried not to show it at home either but I know occasionally I did lash out and yell at [my partner] and that. (Raymond aged 51, maintenance worker)"

Rosslyn’s and Raymond’s accounts illustrate Hoggett’s conception of non-reflexive, or impulsive agency, for by ‘lashing out’ unreflexively they reveal the potential for unintended consequences from the ‘human capacities for destructiveness towards self and others’ [51] p. 37.

While the majority of workers claimed to have experienced stress a small minority had not, and as Rex explained this was because:

"I just sort of accepted the decision. Probably on the day it was announced it didn’t sort of sink in … but even when it did, it didn’t really – maybe I thought about it for an hour one day or something, then I thought ‘oh well we’ve got eighteen months to worry about it’. (Rex aged 60, a trainer)"

For Owen, the ongoing speculation of redundancy blunted rather than intensified the stress of job insecurity because it made it appear more likely. He stated:

"It [speculation] had been going on for probably six, seven years that I can think of. Everyone tells you Mitsubishi is going to close down… We hear about it on the wireless and news before we’d get told at Mitsubishi … So it just got to the stage where you just say ‘oh well if it happens it happens”. I suppose in one way there’d probably be a sense of ease to know what was finally happening. (Owen, aged 56, plant operator)"

This response is typical of the findings of an Australian study on job insecurity [15] which showed that certainty of job status, and even certain redundancy, may be less psychologically damaging than prolonged insecurity. In understanding seemingly contradictory responses to job loss by the same individual, one process worker explained that he had not experienced stress as he was facing a potentially life-limiting illness and was relieved to no longer have to work. Job loss was therefore the lesser of two negative life impacts. Workers’ accounts of stress also highlight a link to agency and changes to perceived control.

### Changes to perceived control

The qualitative analysis of respondents’ accounts of whether job loss had affected their level of perceived control showed that the majority claimed less perceived control. They spoke in terms of ‘pressure’, and ‘no motivation to get up in the morning’. Garth (aged 37) a senior trainer who embraced full time tertiary study never-the-less felt constrained by outside forces. He said ‘I basically lost a lot of control because I had to just turn things upside down and go down a whole new road’. Ken (aged 36), a production worker living with a serious mental illness explained that while he was at Mitsubishi he had a routine, but ‘[now] not having the workplace hanging over my head I don’t have that control to get up in the morning and be motivated’*.* Ken’s account shows how the secure structure provided by employment can be lost following retrenchment [64]. Doreen (aged 59) a canteen worker maintained: 

"I just felt out of control, I just felt like I have got no control over my life anymore … It’s very frustrating looking for work and not getting anything. They [potential employers] don’t even answer your letters. I don’t know how people cope with it."

These accounts by workers who experienced less perceived control often reflected Hoggett’s conception of the self as ‘reflexive object’, in the way in which they were able to reflect on the meaning of their job loss while feeling powerless to overcome the negative impacts [51] p. 50.

Ted (aged 60), a former mechanic, was one of three workers who instead perceived greater control following job loss as he felt he had ‘more control now’ in retirement. Forty five year old process worker Morris was unable to work due to a serious illness, but perceived greater control from ‘no longer having to justify things and be reliable’. When asked about whether job loss had affected his perceived control Michael (aged 39), a maintenance manager and a confident, assured worker who had already experienced a prior redundancy responded:

"No. Not at all. It actually increased it. It made my wife and I a bit more determined to be self sufficient. And it’s driven us in a different tangent now to being dependent on a wage coming in."

Michael explained how he engaged his creative agency to devise strategies to ‘get out’ of the constraints of any future redundancy, stating:

"I mean I’m young, I’m keen and this is the second time in my life I’ve been made redundant. So being made redundant didn’t matter a stuff for me. I’ve got income insurance [protection]. I’ve got my mortgage insured so if I went on the dole well, I’m covered. But I actively engaged in my own employment so that doesn’t happen to me. But a lot of these other people they didn’t do it."

Michael’s account projects Hoggett’s stance of a creative self-as-agent, a person who is an active shaper of personal destiny even if not under the circumstances of his or her own choosing. Michael fits Lister’s typology of a person ‘getting out of’ job loss by employing strategic agency or making longer-term investments to avoid the risks associated with future redundancy. Some other workers perceived they gained greater control from the financial security offered by receiving a carer’s pension, or becoming eligible for employment benefits while able to volunteer in lieu of undertaking paid work.

For respondents who claimed no change to perceived control financial security was a common ameliorating factor. John, a manager for 25 years, expressed a raft of negative outcomes from job loss, but maintained his level of perceived control from the financial security of a redundancy package, cited by many workers as a very enabling structure. Most responses indicating no change to perceived control supported the wider literature showing the links to secure financial status [41]. However, two workers claimed that they maintained control by actively embracing new opportunities, including starting a small business and returning to study after 20 years.

### Loss of self-esteem, shame and loss of status

Self-esteem is the positive self-regard that promotes a sense of approval and success and acts as a spur to agency [65]. While facing her own retrenchment, Sylvia was concerned about her partner’s emotional well-being in the face of his concurrent redundancy. She explained: 

"He lost all his confidence. He feels very unsure of himself and because he doesn’t have any trade skills he feels, not useless, but he can’t find anything other than factory work because that’s all he’s known. (Sylvia, aged 55, production worker)"

Raymond aged 51, a maintenance worker, claimed ‘I was brought up as a breadwinner. I don’t feel like I’m doing that now. So I feel a bit inferior and insufficient sort of thing’. His account supports the research showing the negative impact on men who lose their ‘breadwinner’ role [66] and that people denied the status and respect linked to employment and income become vulnerable to feeling inferior and worthless [10]; or ‘degraded’ as Reg put it: 

"It’s a sense of loss, you know and it’s unhappy to see what people used to do and the skills they had at Mitsi’s [sic] and then see what they’ve degraded to you know; what job roles they’ve got and things like that. Yeah, you can sort of see that they're just all unhappy. (Reg aged 40, section manager)"

Reg experienced loss of self-esteem after accepting a 13 week government-subsidised contract, claiming:

"I’ve gone from a managerial role to more of a baby sitter’s role, so it is probably more demeaning to myself at the moment … I’m not being able to offer the best to the company of what skills I’ve got. So that’s probably changed me a bit."

Wally, aged 63, a vehicle auditor, maintained:

"I think the value of yourself changes, I think you feel as if you’re not as valuable as you used to be but whether that’s old age as well."

David experienced shame from:

"mental pressure, the actual pressure, you can see yourself going downhill through ill health and you’re thinking that they’re going to see that you’re no good anymore. (David, age 57, tradesman)"

In distinction, Ronald claimed to have maintained his self-esteem, even though facing a range of negative outcomes including financial constraints because he had not:

"noticed any [age] discrimination or anything in that area and that is good. In fact a couple of places seem to appreciate experience … And everyone seemed happy with what I do, so I’m quite pleased about that. (Ronald, aged 62, professional worker)"

Ronald’s account supports the claim that self-esteem may be endorsed by feelings of approval and from the success of ‘high attainment’ [65].

### Experiencing a grieving process

The majority of respondents gave affirmative responses to a question about whether they had experienced a ‘grieving process’; expressing it in terms of ‘nostalgia for the environment’, ‘another hit’, a ‘shock to the system’, or having ‘no chance to say goodbye’. One worker felt ‘a pinch in the heart’ while another claimed ‘I can’t get it out of my system’. Tyson displayed a grief reaction when witnessing the decommissioning of equipment after 10 years of his productive labour. He said:

"I was grieving because I nearly ended up just stepping away. I said to my wife it’s really hard, I found it really hard, even to the point of getting a bit tight in the throat side of emotion because when you saw the equipment you just wanted to make an artificial wreath because it got you so frustrated to see all that, and it was like final and it was like ‘wow’. (Tyson, aged 46, production worker)"

However, a minority of workers did not experience grief but instead were glad for other options. Roger, aged 49, a foundry worker for 20 years, stated ‘I was glad to leave and have the opportunity to do something else which was good … I thought it was like a golden opportunity really’. Others were relieved that redundancy had finally happened, or felt that it was time for a change. While in a minority, these workers’ responses supported the literature showing that job loss may eventually be viewed positively, as it can facilitate a change of career and life direction [59].

### Financial strain

No worker stated that they had received a higher income on taking up subsequent employment, and while a few cited comparable income to Mitsubishi, even if tied to more stringent working conditions, most claimed they were earning less; some significantly less. One respondent with a young family and living on Austudy benefits for eligible students was receiving the equivalent of one quarter of his previous wage. Three stated they were earning half their previous wage, a worker who set up a small business was earning AUS $30,000 less per annum, with another earning AUS $20,000 less.

Although choosing the best available employment option, Michael was resigned to accepting a level of pay that was ‘very, very low’ and on leaving Mitsubishi he had to:

"let go all my overtime, I had to let go of my shift allowance, I had to let go of my company car. So I lost, I was on round about $78,000 or $79,000 at Mitsubishi. I’m on $60,000 at [new employment] and haven’t had a pay increase for 15 months. (Michael, age 39, manager)"

Likewise, Richard stated:

"We’re getting a lot less than what we were at Mitsubishi. Mitsubishi had a structured payment for the number of years you’re there and they recognise that and pay you a formula. Nowadays you just contract and it’s a set wage and I’ve gone to monthly wage instead of fortnightly wage. (Richard, aged 45, engineer)"

When asked about their financial status during their second interview, many workers spoke of financial strain, such as needing to ‘tighten up the purse strings’, ‘having less disposable income’, or ‘needing to be more careful’. Workers told of being ‘on a very tight budget’, ‘being 61 and this was not in the plan’, and not sleeping ‘due to worry about bills’. Raymond explained:

"you start becoming frugal like for example using the least electric power as possible. I don’t use my gas oven. Probably the only thing that’s used is when I go and have a shower, that’s the only time the gas is used… I have to be frugal because I’ve got to make the money last as long as possible because it’s going to run out. (Raymond, aged 51, maintenance worker)"

Respondents’ accounts support the conclusion that negative economic consequences of job loss often include considerably reduced wages and earnings in future employment [31]. Those who were in a much worse financial position suggested they were just ‘getting by’ in Lister’s terms because they were ‘trying to weather the storm’ or experiencing a ‘major struggle’. Ken aged 36, a production worker living with a severe mental illness, highlighted the direct link between the financial constraints of job loss and mental health, explaining: 

"Since leaving Mitsubishi I’ve financially struggled and it weighs on your mental state not having that regular income, and it caused a bit more stress."

Respondents’ accounts of financial strain highlighted constraints to their agency, with Angas experiencing financial strain from trying to survive on the basics of life. This was:

"stress in itself because you don’t get a good sleep, you’re thinking to yourself heck I’ve got bills, I mean you still have to pay your bills, still got the electricity bill coming in, and gas bill. You’ve got to register your vehicles and stuff, still got to buy food and things like that, it just goes on and on and on and slowly the money that you have got is depleting. (Angas, aged 55, tradesman)"

Elliott, aged 54, a toolmaker and safety representative, best summarises the workers’ overall financial position, stating ‘I would basically say that money is tighter and [we are] probably struggling more than we were, but we are managing’. These accounts support the wider literature on constraints to agency and social engagement due to precarious employment [12].

## Discussion

In analysing Mitsubishi workers’ experiences of job loss we found a range of negative mental health consequences. We have highlighted some of the complexities of these consequences by adopting a nuanced agency and structure approach. Our broader study was based on a sample of one third (n=371) of the more than 1000 workers facing retrenchment, with our qualitative interviews based on 33 of these 371 respondents. This sample allowed us to conduct in-depth, detailed, qualitative consideration of these workers’ perspectives of job loss. In contrast to other studies that have often focused on one occupational group, this research included accounts from a cohort of vocationally diverse respondents. The discussion is now presented in two main sections: mental health consequences, and agency and structure.

### Mental health consequences

The main mental health consequences of job loss related to increased stress, less perceived control, loss of self-esteem, shame and loss of status, grief or a grieving process, and financial strain. This study adds to the literature on the mental health consequences for workers who will likely experience a more precarious employment environment following job loss. This is associated with the chronic stress which is an important determinant of poor physical and mental health [12]. Mitsubishi workers had been aware for several years that job loss was a real possibility, and we found that for some the stress of uncertainty about retrenchment was lessened by ongoing speculation. However for others, and as confirmed in other research [15], constantly working in a ‘brittle’ environment may gradually increase individual experiences of insecurity and psychological stress. Our findings accord with the view that if restructuring must occur, the period of uncertainty should be kept to a minimum [15].

Our research findings dovetail with other Australian literature linking mass job loss with both emotional and material outcomes [6,67,68] and that the perception of job insecurity impacts on psychological health and well-being [69]. It also reinforces the reality that becoming unemployed is often traumatic due to the conjunction of material and psychological consequences. As the Commission on the Social Determinants of Health (CSDH) states, there is a largely false dichotomy between theories concerned with psychosocial and material factors that influence health, as most material phenomena also have social salience [64]. The mental health findings showing the links between employment conditions, the policy environment, and mental health point to the need for a holistic response to help workers who lose their jobs. This would combine material and economic support with emotional and mental health support, and suggests the need to combine, or consider the interactions between, national policies focusing on employment and industry with those focusing on mental health.

There were examples of workers who found some positive changes to their lives as a result of job loss (and this included some workers who also reported negative consequences). These workers were more likely to be more financially secure, and this reinforces the literature on the mediating role of financial security in the effects of job loss on mental health. Life-stage was also important, with some workers with young children positive about the opportunity to spend more time with their families. The impact of these factors in workers’ lives and the potentially buffering role they might play in adjusting to job loss warrant further investigation.

The complex ways that job loss was experienced by workers and impacted on their mental health reflected the interplay between workers’ agency and broader structural factors. These are discussed below.

### Structure and agency

Adopting a structure and agency approach gave an opportunity to highlight that individual capacity to act in the face of job loss is influenced not only by material and other structural issues, but also by personal or interior factors, individual subjectivities and fluctuating reflexivity. Most workers were reflexive or considered about their circumstances and were able to refrain from expressing impulsive behaviours in the face of job loss. However, accounts of two workers experiencing suicidal ideation, and two workers facing job loss while also responsible for supporting others throughout the redundancy process, showed that people are not always able to contain impulsive or non-reflexive behaviour; including ‘lashing out’ in the face of stressful emotional triggers. This highlights a further level of complexity when theorising the effects of material and emotional consequences of job loss. Taken together, Lister’s and Hoggett’s agency models helped to understand this complexity and also challenge the hegemony of neoliberal economic theory which leads to contemporary policies that place high demands on individual agency while often underplaying structural constraints [70].

A limited number of workers in the study were able to take an optimistic approach to job loss from experiencing enabling or positive lifestyle changes, or finding suitable jobs or employment alternatives; and others had the capacity to employ strategic agency for longer-term benefit by ‘getting out of’ the constraints of job loss through further education and starting their own businesses. However, more often it was (just) ‘getting by’ agency responses that were revealed, and it is important to acknowledge the level of creative agency sometimes required of, and displayed by, people in their struggle to address difficult and complex life challenges. The study revealed a strong perception of many workers ‘being done to’, or the self-as-object, and a particular resonance with traditional, male working-class labour. Unlike self-as-*subject* actors, self-as-*object* actors are ‘unable to impose their will on their environment’ [71] p. 689, and people who are acutely aware of their level of powerlessness ‘teeter on the boundary between anger and despair’ [51] p. 50. As other research has shown, workers experiencing job insecurity may be extremely constrained in their day to day living and their ability to engage in every day social interactions when drawing upon their often limited material and psychological resources [12].

The job losses from the Mitsubishi restructuring also signify the broader shift in employment conditions from those of traditionally stable manufacturing jobs to more insecure arrangements in the services sector [72-74], with implications for workers’ agency. Following retrenchment many respondents in the study took up a range of personal care, cleaning, and other service sector roles. These are usually part time or casual jobs with poor pay, job security, working conditions, and potential career paths [75]. Many were also mature workers who face greater challenges from job loss and who, when compelled to take redundancy as early retirement, express very negative experiences which affects their well-being for years [76]. Our study highlights the ‘wastage’ [24] p. 31 of these more experienced workers when they leave the workplace or find inferior, insecure or temporary employment.

## Conclusion

The capacity to exercise agency in addressing the constraints of job loss must be understood within the context of an individual’s whole of life circumstances, including personal as well as structural factors that together shape these circumstances. Constraints to reflexive agency may lead to workers taking impulsive and negative actions which, in some circumstances, may limit their ability to make positive choices for enabling their health and well-being due to a ‘collapse of agency’ [77] p. 317. This is an important insight with wider implications for health. For example, as behavioural health promotion is both premised on, and focused on, agency and the individual’s capacity to act to improve lifestyle, this invites reflection on the reality that an individual’s lifestyle is not always a ‘choice’, but instead an adaptation made within the context of a wide range of enabling and constraining material structures and differing degrees of individual agency.

Individuals who experience unemployment often carry extra material and psychological burdens associated with other forms of hardship and disadvantage [62] that are further compounded by a new employment context in which they are increasingly intermittently employed and ‘working without commitments’ [14] p. 7. Many retrenched workers, including those from Mitsubishi who lost long term secure jobs, may have accrued greater economic support (including from the relatively generous redundancy packages offered by Mitsubishi) and social support networks compared with those who have been more precariously employed. However, they may not be as attuned to ‘getting out’ of their predicament as workers who are more used to dealing with temporary or precarious forms of employment. They may have also made financial commitments based on a presumption of ongoing job security. The different health impacts and implications for agency between people who lose more secure jobs and those who lose less secure employment suggests a further avenue for research.

Overall our study demonstrates the interaction between the economic and social circumstances that shape job loss and the range of responses that workers can make to that job loss. While there are some very real differences between the workers, the stronger message from our study is the significant mental health impact from the threat of and actuality of job loss.

## Competing interests

The authors declare they have no competing interests.

## Authors’ contributions

FB and AZ led the design of the study, FB, AZ, GJ and LN conducted some of the interviews. All authors were involved in analysis of the data. JA drafted the article and FB, LN, AZ and GJ have critiqued drafts of the paper and their comments were discussed collectively by all authors and used to redraft the paper. The final version of the paper has been approved by all authors.

## Authors’ information

Julia Anaf is a Research Associate in the Southgate Institute for Health, Society and Equity, Flinders University.

Fran Baum is Professor of Public Health and an Australia Research Council Federation Fellow at Flinders University, Adelaide. She is also Foundation Director of the Southgate Institute for Health, Society and Equity & the South Australian Community Health Research Unit. She is a Fellow of the Academy of the Social Sciences in Australia and of the Australian Health Promotion Association. She is a past National President and Life Member of the Public Health Association of Australia. Over her career she has published 129 peer reviewed articles, 9 books, 28 book chapters and 58 reports for government or health organizations.

Lareen Newman works in social health research, taking a critical public health perspective to social determinants, health equity and user access to services. She is increasingly researching communication inequalities and digital technologies as social determinants of health. Lareen previously contributed research for the Commission on Social Determinants of Health and the South Australian Government's Health in All Policies program. She is co-editor of the *Journal of Community Informatics* Special Issue on Health and recent publications include an article in *Community, Work & Family* on the views of automotive workers’ children about the impact of parental redundancy.

Anna Ziersch is a social scientist with a background in social psychology. She is a Senior Research Fellow at the Southgate Institute for Health, Society and Equity and has an overarching interest in health inequities, in particular multidisciplinary and multi-method approaches to understanding the social determinants of health. One of her current areas of focus is researching connections between work and health, and exploring how forms of employment can impact on health. She recently had a paper on the experience of workplace bullying accepted for publication.

Gwyn Jolley completed a M.Sc. (PHC) in 2003 and is currently enrolled as a doctoral candidate by published work. Gwyn’s main research interests are in evaluation of community-based health promotion and primary health care, healthy settings and participatory, qualitative research approaches.

All authors are based at the Southgate Institute for Health, Society and Equity, Flinders University, Adelaide, South Australia.

## Pre-publication history

The pre-publication history for this paper can be accessed here:

http://www.biomedcentral.com/1471-2458/13/110/prepub
